# Prediction of Disruptive Behavior over Time from Changes in Patients’ Global Functioning in Acute Psychiatric Care

**DOI:** 10.1007/s10488-024-01355-5

**Published:** 2024-03-23

**Authors:** Tamar de Boer, Marijn Pietersma, Bea Tiemens

**Affiliations:** 1 Pro Persona Pompestichting, Nijmegen, The Netherlands; 2 Pro Persona Research, Wolfheze, The Netherlands; 3 Behavioural Science Institute, Radboud University, Nijmegen, The Netherlands

**Keywords:** Disruptive behavior, Brøset Violence Checklist, Kennedy Axis V, Risk assessment, Early intervention, Acute psychiatric care

## Abstract

Disruptive behavior of patients in acute psychiatric care is a problem for both patients and staff. Preventing a patient’s impending disruption requires recognizing and understanding early signals. There are indications that a change in a patient’s global functioning may be such a signal. The global functioning of patients is a multidimensional view on their functioning. It captures a patient’s psychological symptoms, social skills, symptoms of violence, and activities in daily living. The aim of this study was to gain insight into the predictive value of global functioning on the risk of disruptive behavior of patients in acute psychiatric care. Also assessed was the time elapsed between the change in global functioning and a patient’s disruptive behavior, which is necessary to know for purposes of early intervention. In a longitudinal retrospective study, we used daily measurements with the Brøset Violence Checklist (BVC) and the Kennedy Axis V (K-As) of each patient admitted to two acute psychiatric units over a period of six years. Data from 931 patients for the first 28 days after their admission were used for survival analysis and cox regression analysis. Disruptive behavior was mostly observed during the first days of hospitalization. Global functioning predicted disruptive behavior from the very first day of hospitalization. A cut-off score of 48 or lower on the K-As on the first admission day predicted a higher risk of disruptive behavior. If functioning remained poor or deteriorated substantially over three days, this was an additional signal of increased risk of disruptive behavior. Improvement in global functioning was associated with a decreased risk of disruptive behavior. More attention is needed for early interventions on global functioning to prevent disruptive behavior.

## Introduction

One of the challenges in acute psychiatric care is preventing or reducing disruptive behavior. Disruptive behavior is a combination of aggressive and violent behavior and symptoms. It includes aggressive behavior, such as spitting, scratching, or pinching, but also violent behavior, such as using physical force by slapping, punching, kicking, or biting, or the use of an object as a weapon. Furthermore, verbal threats involving no physical contact may also be classified as disruptive behavior (Clarke et al., 2010). Between 3% and 44% of patients in acute psychiatric care are aggressive during their hospitalization (Grassie et al., 2001; Lockertsen et al., 2021; Lozzino et al., 2015). Disruptive behavior has negative effects on both patients and staff (Dack et al., 2013), and it leads to anxiety and insecurity in other patients (Hamrin et al., 2009).

Disruptive behavior is a complex and dynamic phenomenon arising from different patient-related risk factors. Patients diagnosed with psychosis, a personality disorder, or autism are at higher risk for disruptive behavior than other patients (Brendel et al., 2010; Steinert & Whittington, 2013; Krippl & Karim, 2011; van de Sande et al., 2013a). Situational circumstances can also trigger disruptive behaviors (Faay et al., 2012; Steinert & Whittington, 2013). In fact, many violent outbursts are preceded by frustrations or restrictions, often imposed by mental health professionals (Duxbury et al., 2008; Steinert & Whittington, 2013). Misperceptions of situations and a misunderstanding of another person’s intentions and attitude might also trigger disruptive behavior (Steinert & Whittington, 2013). The nature of the connection between a patient’s psychiatric diagnosis and disruptive behavior is unclear; nevertheless, disruptive behavior is likely to increase when multiple risk-factors are present.

Understanding a patient’s previous signals of impeding dysregulation can help in recognizing and preventing disruptive behavior. There are indications that a change in a patient’s global functioning may be such a signal (van de Sande et al., 2013b, 2017). Global functioning captures four domains of symptoms and skills (Kennedy, 2003), including psychological impairment, such as psychotic symptoms, motivation and mood disturbance, and social skills, such as interpersonal skills, communication skills, and awareness of social norms. Further, it entails symptoms of violence, such as threatening, suicidal, or sexually violent behavior. A final domain of global functioning includes activities in daily living and occupational skills, such as job skills, self-care skills, and personal hygiene (Kennedy, 2003). A patient’s global functioning can fluctuate strongly and can vacillate within a matter of hours (Steinert et al., 2007b). Finally, changes in one of the domains of a patient’s global functioning can be a precursor of subsequent disruptive behavior.

There are multiple observational tools that mental health professionals can use to focus on patients’ disruptive behavior and global functioning. In the High and Intensive Care Units of acute psychiatric care in the Netherlands, it is mandatory that the Brøset Violence Checklist (BVC) be used, and it is advised that the Kennedy Axis V (K-As) (van Mierlo et al., 2013) also be used. The BVC is an observational instrument that measures (a) three types of disruptive behavior (verbal threats, physical threats, and violence against objects), and (b) three kinds of symptoms (confusion, irritability, and noisiness) (Clarke et al., 2010; van de Sande et al., 2013a). The K-As is an observational tool with four subscales: psychological functioning; social functioning; violence (toward self or others); and activities of daily living (ADL), including occupational skills (Kennedy, 2003). Together, the four subscales capture a patient’s global functioning (Kennedy, 2003). Previous studies have investigated the association between global functioning and coercion (van de Sande et al., 2013b, 2017; Partridge & Affleck, 2018). Although there is no research on the direct association between global functioning and disruptive behavior, early detection of an increased risk of disruptive behavior based on changes in global functioning might provide an opportunity to prevent disruptive behavior from occurring.

### Current Study

The aim of this study was to gain insight into the predictive value of global functioning on the risk of disruptive behavior among patients in acute psychiatric care. The research question was: Can changes in the global functioning of patients in acute psychiatric care predict their risk of engaging in disruptive behaviors? In acute psychiatric care, assessment of the risk of disruptive behaviors must take place in time for interventions to be deployed to prevent an escalation. Therefore, the time that elapsed between the change in global functioning and the patient’s disruptive behavior was also assessed. Different aspects of patients’ global functioning might have different associations with their disruptive behavior. For instance, in a longitudinal study, van de Sande et al. (2017) studied the association between the K-As and seclusion and found dysfunctional scores on the subscales psychological functioning, social skills, and violence (to self/others) during the week of seclusion. The subscale violence (to self/other) was significantly positively associated with patients being placed in seclusion (van de Sande et al., 2017). This outcome was expected, but in another study (van de Sande et al., 2013a) deteriorated psychological and social functioning were also significantly associated with occurrences of seclusion. The association between global functioning and seclusion suggests that the global functioning subscales might be associated with disruptive behavior and that they might be able to predict the occurrence of disruptive behavior in the future. To select the best predictors of disruptive behavior, we aimed to determine how well changes in each of the four subscales of global functioning predicted the risk of future disruptive behaviors.

## Method

The design of the current study was retrospective longitudinal. The study was conducted in a large mental health organization in the eastern part of the Netherlands. Routine anonymized data were used from patients in two different acute psychiatric units. The data included daily measurements using the Brøset Violence Checklist (BVC) and the Kennedy Axis V (K-As). The mental health professionals were trained to use these observational measurements as the basis for their daily report in the *Crisis Monitor*. Twice a day, once in the afternoon and once in the evening, different mental health professionals working on the acute psychiatric units monitored all patients who had been admitted to each unit.

### Sample

Data from Unit 1 encompassed the period from 2016 to 2018 and from Unit 2, the period from 2016 to 2022. Data from all patients who were admitted for a minimum stay of seven days were included. Patients who were younger than 18 years or older than 65 years were excluded because they should have been in a unit for juveniles or older adults, respectively. Data from only the first 28 days of patients’ hospitalization were included in order to reduce the proportion of missing values from the K-As. The final sample consisted of 931 patients whose demographic and clinical characteristics are shown in Table 1. It shows that 502 patients (53.9%) were male and 429 (46.1%) were female. Patients’ mean age was 37.74 (*sd* = 12.00) years. Because van de Sande et al. (2013a) found different risks of disruptive behavior among younger and older patients (with a cutoff at 35), our sample was divided into two groups: 35 years and younger and older than 35 years. The sample was categorized according to Van der Molen et al.’s (2007) into five most commonly used DSM diagnostic groups, with psychotic disorder as the most common.

**Table 1:** Patients’ characteristics (*N* = 931)

	N	%
*Sex*		
Male	502	53.9%
Female	429	46.1%
*Age*		
Age ≤ 35	448	48.3
Age > 35	483	51.7
*Main diagnosis*		
Mood or anxiety disorder	279	30.0%
Psychotic disorder	446	47.9%
Personality disorder	92	9.9%
Neurobiological development disorder	45	4.8%
Other disorder	62	7.4%

### Instruments

The Brøset Violence Checklist (BVC) is a validated observational instrument for measuring disruptive behavior of patients in acute psychiatric care. It is specifically designed to assess the first signs of aggression and violence (van de Sande et al., 2013a). The BVC is an observational instrument that measures (a) three types of disruptive behavior (verbal threats, physical threats, and violence against objects), and (b) three kinds of symptoms (confusion, irritability, and noisiness) (Clarke et al., 2010; van de Sande et al., 2013a). In this study, only the three items of disruptive behavior were used. The BVC has an moderate interrater reliability (*k* = 0.44) (Almvik et al., 2000). In the current study, the internal consistency of the BVC was questionable, with a Cronbach’s alpha of 0.62 for the first measurement.

Patients’ global functioning was assessed using the Kennedy Axis V Short Version (K-As). This questionnaire includes these subscales: psychological functioning, social functioning, violence (to self/others), activities of daily living, and occupational skills (ADL). The subscales capture the clinician’s impression of the patient’s overall current level of functioning during the day (Kennedy, 2003). Functioning in the individual domains is scored in steps of 5 points, using 40 different descriptions of the degree of good or problematic functioning, 10 per subscale. The scores linked to the descriptions increase in steps of 10, with 5 as the lowest score and 100 as the highest (van de Sande et al., 2017). A score greater than 50 indicates strength in the patient’s functioning, whereas a score of 50 or less indicates problematic functioning (Kennedy, 2003). Patients with a score of 50 or less on global functioning are often judged to be in need of hospitalization. The K-As has substantial interrater reliability (0.79), which was calculated using the intraclass correlation coefficient and Person’s *r* (Faay et al., 2012). In the current study, internal consistency measured with Cronbach’s alpha was 0.86 for the first measurement.

### Statistical Analysis

Data preparation and analysis were performed using R (Version 4.3.2). A database was constructed that contained the highest daily scores from the Brøset Violence Checklist (BVC) and the lowest daily scores on the Kennedy Axis V (K-As). A high score on the BVC indicates disruptive behavior, and a low score on the K-As indicates poor global functioning. A threshold score of one or higher on the disruptive behavior items of the BVC was used to determine the occurrence of a disruptive event. For each patient, measurements taken from the first day until the 28th day of hospitalization were used for the analysis, and the first day on which disruptive behavior occurred was identified and flagged as an event. Patients who did not experience any disruptive event during the 28 days of hospitalization were censored. However, the last day of their hospitalization was flagged for further analysis. Patients’ sex, age, main diagnosis, and time of hospitalization obtained from the hospital’s admission database were also included.

Since the K-As also includes a subscale for ‘violence’, whose scores are part of the global score, this could strengthen the predictive value of the K-As. Therefore, all analyses were also conducted without the violence subscale. However, since in practice the total global score is used, the results of the total score are given unless the total score without the violence subscale show different results.

#### Probability of Disruptive Behavior

The occurrence of disruptive behavior was first explored using Kaplan Meier survival curves (R Package Survival, Version 3.5-7). Interactions among patients’ age, sex, and diagnosis were examined using Cox proportional hazards regression analysis (R Package Survival, Version 3.5-7).

#### Predictive Value of Global Functioning on First Admission Day

The total K-As score for global functioning from the first day of admission was used to examine the relationship between the total K-As score on the day of admission and the time to disruptive behavior.

#### Cut-Off for Prediction on First Day of Admission

To determine whether a signal of increased risk of disruptive behavior could be given as early as the first day of admission, a Receiver Operating Characteristic (ROC, R-Package pROC, Version 1.18.0) curve was plotted. With use of the ROC curve, the ability of the K-As score on the first day of admission to predict a disruptive event (yes/no) was assessed. This was done to evaluate the sensitivity, specificity, positive and negative predictive value of the K-As score as the best predictor of the event. The total K-As score of first admission day, categorized as below or above the cut-off score, was used to examine the relationship between the dichotomized total K-As scores at day of admission and time to disruptive behavior.

#### Predictive Value of Change in Global Functioning in the Three Days Prior to Disruptive Behavior

For each patient with an event, the three days leading up to the flagged day of an event were examined. The K-As scores were categorized as follows:

- If a patient’s score on the K-As increased by 10 points or more (the score entered at least a higher descriptive category) during the three days before the event, this was categorized as *Improving*.
- If a patient’s score on the K-As decreased by 10 points or more (the score entered at least a lower descriptive category) during the three days before the event, this was categorized as *Worsening*.
- If a patient’s score on the K-As did not change by 10 points or more during the three days before the event, and the total K-As score on the day of the event was equal to or lower than the cut-off score, this was categorized as *No Change < = cut-off*.
- If a patient’s score on the K-As did not change by 10 points or more during the three days before the event and the total K-As score on the day of the event was higher than the cut-off score, this was categorized as *No Change > cut-off*.

These four categories were used in a Kaplan-Meier survival analysis to examine the relationship between the change in global functioning across time before the event. Additionally, the same categorization was used with each subscale on the K-As. However, if a patient did not change by at least 10 points, the total K-As score was still examined to determine whether the patient’s score had been categorized higher or lower than the cut-off score, instead of examining the score on that subscale. The categories for each subscale were utilized in separate Kaplan-Meier survival analyses to explore how the change in functioning on each subscale during the three days leading up to the event was related to time to the event.

#### Predictive value of global functioning on the first day of admission and changes in global functioning

To determine whether change in global functioning provides additional information about the global functioning score on admission day, a Cox proportional hazards regression analysis was performed. Age, sex and diagnosis (dichotomized as psychosis yes/no) of patients were added to examine any influence of these patient characteristics. A second Cox proportional hazards regression analysis also examined interactions with these patient characteristics.

#### Predictive Value of Day-to-Day Changes in Global Functioning Prior to Disruptive Behavior

To examine daily changes in K-As scores leading up to the flagged day, difference scores from one day to the next were calculated. The difference between the K-As score on the flagged day and each prior day until seven days before the flagged day had been reached were calculated. Each difference score was placed into one of four categories based on its magnitude and relationship to the corresponding K-As value. The categories were as follows:

- Improving: Difference scores with an increase of five or more points on the K-As.
- Worsening: Difference scores indicating a decrease of five or more points on the K-As.
- No Change < = cut-off: Difference scores smaller than five, but with a total K-As score equal to or lower than the cut-off score.
- No Change > cut-off: Difference scores smaller than five, but with a total K-As score higher than the cut-off score.

These categories were then used in a Cox proportional hazards regression analysis to determine relationships among the changes in the K-As scores prior to the disruptive event. Patients who experienced a disruptive event earlier than the day of the difference scores calculation were excluded from those analyses. Patients with missing K-As scores were also excluded from the analysis.

## Results

**Fig. 1:**
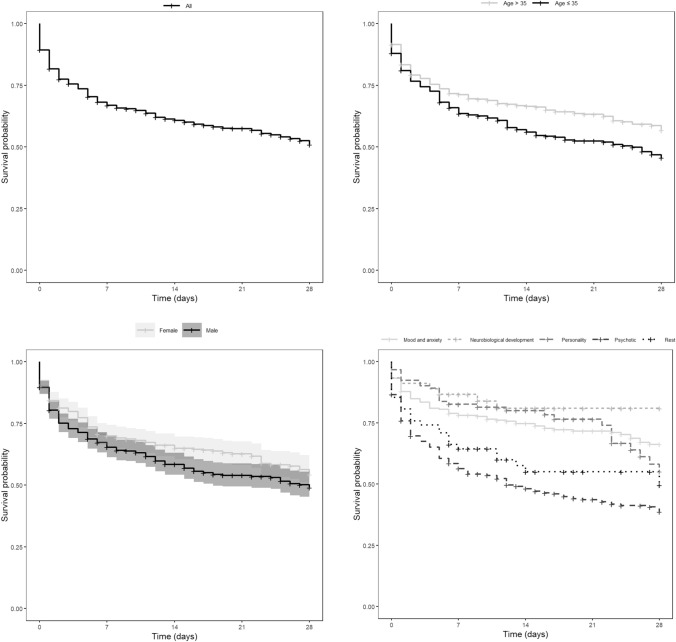
**a** The survival probability of the disruptive behavior over time (*N* = 931). **b** The survival probability of the disruptive behavior of two age groups over time (*N* = 931). **c** The survival probability of the disruptive behavior of two gender groups over time (*N* = 931). **d** The survival probability of disruptive behavior of five diagnostic groups over time (*N* = 931)

Of the 931 patients, 404 (43.4%) displayed disruptive behavior during their first 28 days of their hospitalization. Figure 1a shows that most events took place at the beginning of the hospitalization. Patients’ age on the first day of hospitalization was significantly (*p* = .01) associated with the risk of disruptive behavior over time (*HR* = 0.99; 95% *CI*, 0.98 − 1.00). Patients aged 35 or younger had a lower survival probability of disruptive behavior than patients older than 35 at almost all time points (*HR =* 1.35; 95% *CI*, 1.11–1.55; *p* < .01) (Fig. 1b.). Male patients had a lower survival probability of disruptive behavior than female patients (*HR =* 1.22; 95% *CI*, 1.00–1.50; *p* = .05) (Fig. 1c.). Patients with a psychotic disorder had the lowest survival probability at almost all time points (*HR* = 2.36; 95% *CI*, 1.84–3.04; *p* = < 0.01) (Fig. 1d.). Patients with a neurobiological developmental disorder had the highest survival probability.

### Predictive Value of Global Functioning on First Day of Hospitalization

Data from 723 patients with 274 events were analyzed. Better global functioning on the first day of hospitalization was significantly associated with a lower risk of disruptive behavior over time (*HR* = 0.95; 95% *CI*, 0.94-0.95). The cut-off point with the best combination of sensitivity and specificity for predicting disruptive behavior was 48 (*Se* = 0.66; *Sp* = 0.76), which is close to the cut-off of 50 indicating problematic functioning. The AUC = 0.75 (95% *CI* 0.71-0.79) and the positive and negative predictive values were 0.62 and 0.79, respectively. Patients with a score of 48 or lower on first day of hospitalization had a lower survival probability of disruptive behavior than patients with a score higher than 48 (HR = 4.49; 95% CI, 3.49–5.78; *p* < .01).

### Predictive Value of Changes in Global Functioning within Three Days Prior to Disruptive Behavior

**Fig. 2:**
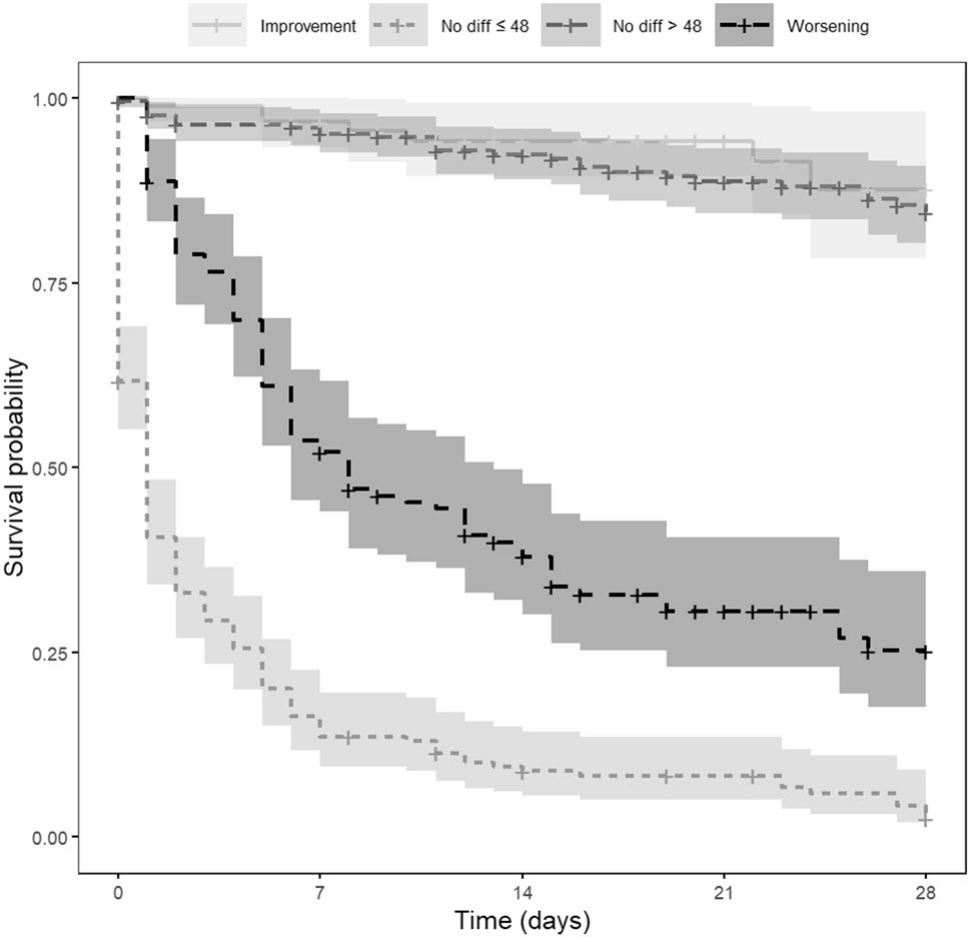
Survival probability of changes in global functioning including the cut-off point (*N* = 664)

Figure 2 shows the predictive value of four patterns of changes in global functioning, with stable global functioning divided into a score of 48 or less and higher than 48. Patients whose global functioning improved (reference category) and patients with a score higher than 48 at the day of the event and stable the days before, were less likely to exhibit disruptive behavior during the next three days. In contrast, patients with a global functioning score of 48 or lower on the day of the event who were stably low the days before, and those who deteriorated on global functioning, had a significantly increased risk of disruptive behavior (*HR* = 39.75 *CI*, 18.61–84.91 and *HR* = 12.99 *CI*, 6.00–28.08).

**Fig. 3:**
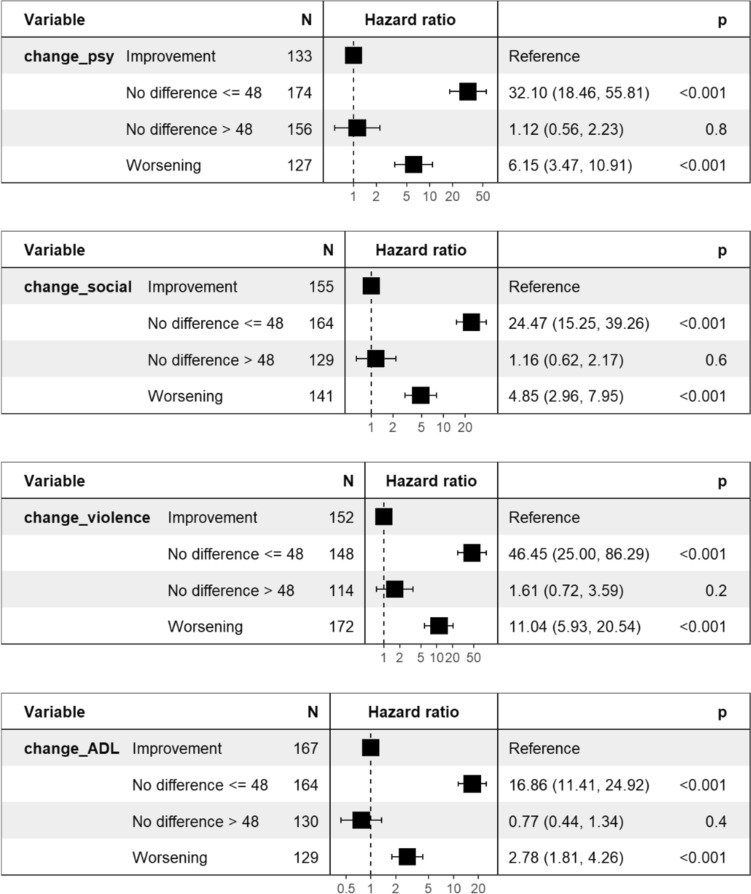
Hazard ratios for the association between changes in the subscales of global functioning and disruptive behavior

Figure 3 shows the hazard ratios for the association between changes in the global functioning subscales for disruptive behavior. The reference pattern in the analyses was *improvement*. A stable functioning of 48 or less on the four subscales of global functioning was consistently associated with a much higher risk of disruptive behavior within three days, as indicated by hazard rations ranging from 16.86 to 46.45.The *worsening* patterns on all subscales were also consistently associated with an increased risk of disruptive behavior within three days, as indicated by hazard ratios ranging from 2.78 to 11.04.

**Fig. 4:**
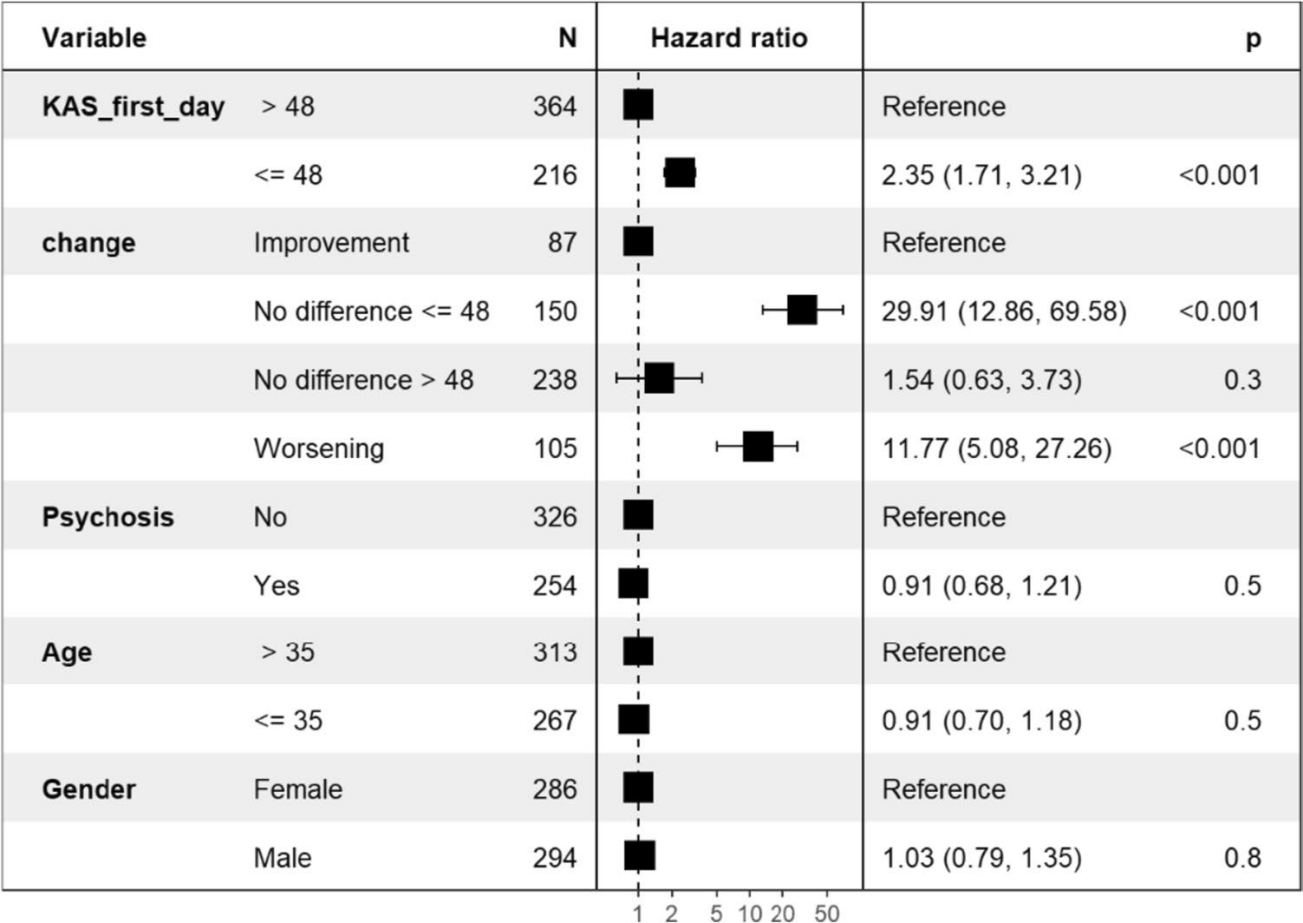
Hazard ratios for the association between score on global functioning on the first day, changes in global functioning, diagnosis, age, gender and disruptive behavior

To determine whether change in global functioning provides additional information about the global functioning score on admission day, a Cox proportional hazards regression analysis was performed. Figure 4 shows the Cox proportional hazards model with the dichotomized score on global functioning on the first day of hospitalization, the pattern of change, diagnosis (dichotomized as psychosis yes/no), age and gender as independent variables. In addition to a low score on the first day, patients who maintained a stable low score on global functioning or patients who deteriorated had an additional increased risk of disruptive behavior. No additional main effect of diagnosis, age of gender was found.

**Fig. 5:**
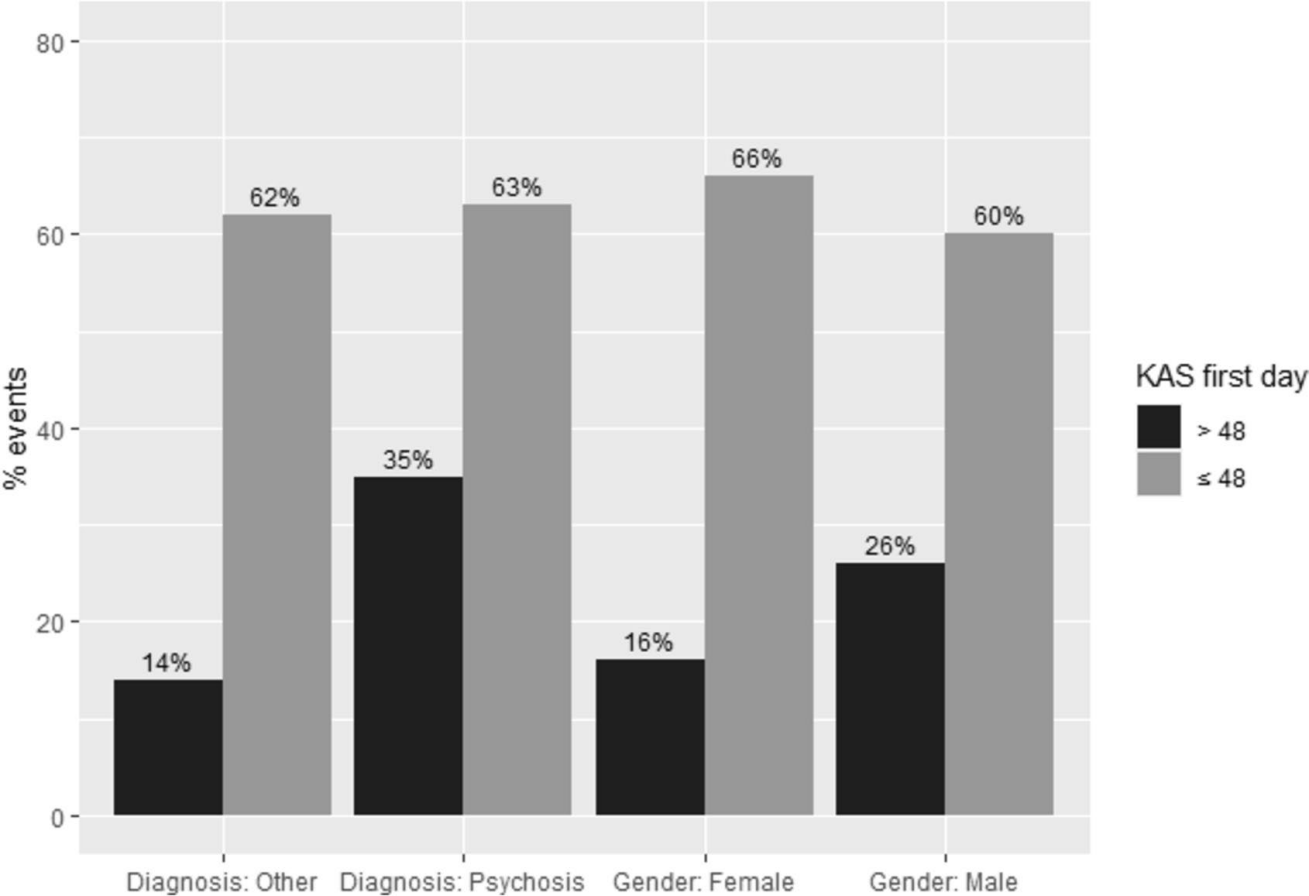
Percentage of events for patients with global functioning on the first day > 48 or ≤ 48, distinguished by patients with psychosis or another diagnosis and by men and women

Interaction effects were found for global functioning on the first day of admission with a diagnosis of psychosis versus other diagnosis (*HR* = 0.36 *CI*, 0.18 − 0.69) and with gender (*HR* = 0.47 *CI*, 0.26 − 0.88). For patients with a diagnosis of psychosis, the hazard ratio for the interaction was lower than for patients with another diagnosis in that for patients with a K-AS score less than or equal to 48, there was little difference between these groups in the percentage who engaged in disruptive behavior (63% and 62%, respectively; see Fig. 5). In patients with a K-AS score higher than 48, this difference was greater (35% and 14%, respectively). Thus, with better global functioning, patients with a diagnosis of psychosis were more likely to engage in a disruptive event. This difference disappeared when global functioning was poor. Figure 5 also shows the interaction effect for gender. With better functioning, men had a higher risk of a disruptive event than women, while women had a higher risk when global functioning was poor on the first day.

### Predictive Value of Day-to-Day Changes in Global Functioning in the Days Prior to Disruptive Behavior

A stable global functioning of 48 or less on one day compared to the previous day and across seven days was associated with a higher risk of disruptive behavior, with HRs of 2.80 to 15.39. Worsening in global functioning was associated with a higher risk of disruptive behavior only on the day of the event, in contrast to the day before the event (*HR* = 13.96; 95% *CI*, 6.80-28.65). Stable global functioning greater than 48 on Day One compared with Day Two before the event was associated with a lower risk of disruptive behavior (*HR* = 0.50; 95% *CI*, 0.30-0.83).

### Analyses without the ‘violence’ Subscale of the K-AS

All results of the analyses with the global score without the violence subscale were similar. Only some Hazard Ratios were slightly lower.

## Discussion

The aim of this study was to examine the predictive value of global functioning on the risk of disruptive behavior in patients in acute psychiatric care. The ability to predict disruptive behavior based on changes in global functioning would provide the opportunity to prevent the disruptive behavior. Doing so could improve the health, safety, and wellbeing of both patients and staff. To determine the predictive value of global functioning, a longitudinal database was used that included data from the first 28 days of hospitalization of 931 patients from two acute psychiatric units.

The disruptive behavior was observed mostly in the first days of patients’ hospitalization. A cut-off score of 48 or lower on the K-As on the first admission day predicted a higher risk of disruptive behavior. In addition, the course of patients’ global functioning appeared to have predictive value for disruptive behavior. Patients with stable poor global functioning were at highest risk of disruptive behavior. Improvement in global functioning was associated with a decreased risk of disruptive behavior. Disruptive behavior could be predicted one day before it occurred by a decline of 5 points in a patient’s global functioning or of 10 points over three days prior.

### Occurrence of Disruptive Behavior and Patients’ Characteristics

Of the 931 patients assessed, 404 (43.4%) of them showed disruptive behavior during the first 28 days of their hospitalization. This percentage is much higher than previously found, such as in Lozzino et al.’s (2015) study, in which nearly one in five patients admitted to acute psychiatric care showed some form of disruptive behavior during their hospitalization. In the present study, some patient characteristics were related to a greater likelihood of disruptive behavior. A factor that was associated with a higher risk of disruptive behavior was patients’ age. Specifically, patients younger than 35 were significantly more likely to have a disruptive event. This is consistent with van de Sande et al. (2013a) study, in which patients younger than 35 were significantly more likely to be placed in seclusion and with Barlow et al.’s (2000) study, in which patients who were younger than 32 and actively psychotic were at high risk for being aggressive.

Also in the current study, patients diagnosed with a psychotic disorder were at higher risk of disruptive behavior during their hospitalization than patients diagnosed with another kind of disorder. However, we found this difference mainly in the group of patients who showed good global functioning on the first day of hospitalization. If functioning was poor, the risk of disruptive behavior was high for all patients.

In the current study, we also found d that patients’ gender was related to their risk of engaging in disruptive behavior. Bonta et al., as cited by Steinert and Whittington (2013), described being male as one of the strongest individual predictors of violent behavior. In addition, van de Sande et al. (2013a) found a greater likelihood of male patients being in seclusion, and Lockertsen et al. (2021) found that females were significantly less likely than males to be violent. In the current study also for gender an interaction effect was found with global functioning on the first day. With better functioning, men had a higher risk of a disruptive event than women, while women had a higher risk when global functioning was poor on the first day.

### The Timing of Disruptive Behavior

This study found that disruptive behavior was most commonly observed at the beginning of patients’ hospitalization. This is consistent with Sarver et al.’s (2019) study in which BVC scores of two or higher were usually observed during the first three days after patients’ admission. Patients are admitted to acute psychiatric units because they are in crisis. Thus, it would appear likely that disruptive behaviors are more common at the beginning of patients’ hospitalization. In this stage of the hospitalization, little information is available about the patient.

### The Value of Global Functioning for Predicting Disruptive Behavior

The current study showed that patients with the lowest global functioning scores were more at risk of engaging in disruptive behavior during their hospitalization. In fact, global functioning on the first day of hospitalization was significantly associated with the risk of disruptive behavior across time. To be able to offer more guidance for practitioners, we conducted a ROC analysis, which yielded a cut-off score of 48, below which the risk of disruptive behavior was significantly increased. The cut-off of 48 is close to the 50 or below which, according to Kennedy (2003), indicates problematic functioning. In their researchVan de Sande et al. (2013a) advised using a cut-off score of < 30 for psychological/social functioning and < 50 for violence (to self/others). In the current study, only the cut-off scores for total global functioning were analyzed, but a score of 48 or less should increase the alertness of mental health professionals to the possibility of preventing potentially disruptive behavior. However, Kennedy (2003) recommended using individualized cut-off scores in order to best tailor interventions for the individual patient.

Global functioning seems to predict disruptive behavior from the very first day of hospitalization. If functioning remains poor or deteriorates substantially over three days, this is an additional signal of increased risk of disruptive behavior. These findings suggest that there is an opportunity to control disruptive behavior at an early stage (Faay et al., 2012; Steinert & Whittington, 2013). However, functioning can also fluctuate within a matter of a few hours (Steinert et al., 2007). Therefore, we compared the predictive value of an improvement or a worsening in global functioning with stable global functioning. The results showed that an improvement in global functioning was associated with a reduced risk of disruptive behavior within the next three days. Thus, improving global functioning could potentially help prevent disruptive behavior at an early stage.

The question we posed, however, was whether it would be better to focus mainly on patients whose functioning deteriorated or also on patients whose functioning remained stable. The predictive value of stable dysfunctioning was found to be different from both stable good functioning and stable poor functioning. Patients with stable good functioning had a lower risk of disruptive behavior, whereas patients with stable poor functioning had a higher risk. Thus, in the latter group, possibly improving functioning would lower the risk of patients’ engaging in disruptive behavior. In Theruvath-Chalil et al.’s (2020) study, staff proactively intervened when a patient’s BVC score was greater than or equal to three, in their attempt to reduce the risk of disruptive behavior. Theruvath-Chalil et al. (2020) analyzed a wide range of risk reduction strategies, including the use of medication *as required*, interpersonal interventions, and the management of environmental contingencies. However, additional non-restrictive interventions were planned. Staff responded better to patients’ behavior before it escalated to the point where it became necessary to apply physical interventions and associated restrictions (Theruvath-Chalil et al., 2020).

The results for global functioning were also found on the four subscales in our study. This is in line with the finding that risk assessments should include both predictors of aggression and patients’ mental health status (van de Sande et al., 2013a). Van de Sande et al. (2013a) suggest that improving the level of patients’ psychological and social functioning could lead to fewer coercive actions.

### Research Implications and Recommendations

To improve the health, safety, and wellbeing of both patients and staff, disruptive behavior should be minimized. Previous studies have revealed that disruptive behavior needs to be controlled at an early stage (Faay et al., 2012; Steinert & Whittington, 2013). Therefore, better integration of results from the BVC and the K-As daily reports is needed. Using these instruments on a daily basis during hospitalization can assist mental health professionals in analyzing changes over time and predicting disruptive behavior at an early stage. This would also provide more information for tailoring individualized treatment plans in order to prevent disruptive behavior. In the high intensity care (HIC) units within acute psychiatric care in the Netherlands, it is mandatory that the BVC be utilized, and it is advised that the K-As also be used. This is based on the assumption that doing so would help to insure the safety of both patients and staff (van Mierlo et al., 2013).

The treatment utilized within the HIC is focused on recovery-oriented care, which means that it focuses on recovery of patients’ health, daily functioning, societal roles, and identity. Medication (which is sometimes compulsory), psychoeducation, network involvement, the use of experienced experts, and art therapy are components of the treatment (van Mierlo et al., 2013). However, not every HIC has all these treatment components available, and some HIC specializes in a specific type of the treatment. The findings of the present study suggest that treatment aimed at strengthening global functioning might prevent patients from engaging in disruptive behavior. This is consistent with the various guidelines for the treatment of a psychosis, which state that in addition to clinical, psychological, recovery, the care system should support patients’ personal and social recovery from the beginning of their treatment (AKWA, 2017; NICE, 2016).

In the current study, patients with either stable low or deteriorating social functioning were much more likely to exhibit disruptive behavior within the next three days. Impaired social functioning means that there are limitations on such patients’ interpersonal communication skills, that they lack awareness of social norms or sexually appropriate behavior (Kennedy, 2003). To improve a patient’s social functioning, several interventions are available. Group engagement contributes to better social functioning in patients with a personality disorder or dispositional connectedness (Kealy et al., 2020). Mindfulness-based programs, such as the SocialMIND-8, improve social cognition, attributional style, and self-care abilities of patients with psychosis (Mediavilla et al., 2021). The components of the SocialMIND-8 program include awareness of the present moment, the ability to mentalize, coping with distress, radical acceptance, unconditional friendship and compassion, cultivating wholesome relationships, and living a balanced lifestyle (Mediavilla et al., 2021). Ultimately, continuity of care and thus a stable therapeutic relationship might improve patients’ social functioning (Theodoridou et al., 2015). To improve the social functioning of patients in acute psychiatric care, a mental health professional should have a strong investment in the therapeutic relationship, support patient in their efforts to mentalize and use coping skills, and encourage patients to engage in the group.

Patients with worsening or stable low violence risk were more likely to exhibit disruptive behavior in the next three days. Patients might be impaired by their violence to self or others. Such patients might be threatening or assaultive, suicidal, homicidal, or sexually violent (Kennedy, 2003). Inpatient psychiatric staff can decrease patients’ potential for exhibiting violence by using therapeutic relationship strategies, such as good communication skills, advocating for clients, being available, having strong clinical assessment skills, providing patient education, and collaborating with patients in their treatment planning (Hamrin et al., 2009). Cohen et al.(2021) recommend long-term psychotherapy for patients with chronic risk factors, cognitive restructuring for the suicidal narrative, and medication to reduce arousal of patients with the suicide crisis syndrome. Crisis intervention can be necessary to insure safety, such as restricting the means to inflict harm (e.g., removing access to weapons) (Cohen et al., 2021). To improve the violence (to self/other) of patients in acute psychiatric care, a mental health professional can invest in the therapeutic relationship, collaborate with patients in treatment planning, and identify patients who are at high risk of suicidal behavior by using cognitive restructuring to deal with the suicidal narrative. Some HIC units have special rooms for patients at high risk of behaving suicidally. These rooms are designed without access to any objects that could be used as a weapon for patients to hurt themselves.

Patients with a low stable or worsening ADL were more likely to engage in disruptive behavior within the next three days. Patients whose ADL is impaired might have poor job skills, lack self-care skills, have poor workmanship, lack basic survival skills, or have poor hygiene (Kennedy, 2003). Van Mierlo et al. (2013) suggest that movement, such as that involved in sports or simply walking, can help patients to cope with tension. Also, including patients in daily household chores is important for helping them to stay connected with others in their daily living (van Mierlo et al., 2013). Thus, continuing to support the ADL of patients in acute psychiatric care can prevent disruptive behavior from occurring. For this reason, there should be a daily program that keeps patients moving and includes them in the activities of daily living.

No single intervention or set of interventions will act as a panacea for reducing conflicts and containing patients, so that a number of different strategies will be needed (Theruvath-Chalil et al., 2020). We hypothesize that improving patients’ social functioning and their ADL and reducing their violence tendencies might prevent them from engaging in disruptive behavior. It is, however, currently unknown which interventions would be best for achieving this improvement. A better understanding of the mechanisms involved in the prevention of disruptive behavior through the use of interventions focused on patients’ functioning is needed. Further research is also needed to study the preventive value of different interventions for improving patients’ social functioning and their ADL and reducing their violence tendencies.

### Strength and Limitation

To our knowledge, this was the first study to examine the relationship between patients’ level of functioning and their commission of disruptive behavior. In choosing which analyses to use, we were always thoughtful of the implications for clinical practice. This was a clear strength of the study; nevertheless, the study also had an obvious limitation.

A clear limitation was the many missing values on the Kennedy Axis V (K-As) because many patients had to be excluded. The data from Unit 2 was for the period from 2016 to 2022. In the last year, this Unit stopped using the K-As because of changes in the mental health professionals who were employed, a lack of training in the use of the K-As, and the perception that little was derived from using the K-As. As a result, a portion of the patients from the last year of the study had to be removed from the sample. Nevertheless, the number of data points that were filled was reasonably high. Longitudinal studies in similar settings lasted for only one year (Lockertsen et al., 2021). By contrast, this study utilized daily assessments across three years from one of the acute psychiatric units and across six years from the other unit. This was a considerable strength of the study.

### Conclusions

The current study focused on the value of changes in global functioning for predicting the occurrence of disruptive behavior among patients in acute psychiatric care. The goal was to be able to tailor individualized treatment plans in order to prevent disruptive behavior from occurring. The study showed that patients with the lowest global functioning were at higher risk of engaging in disruptive behavior during their hospitalization. A cut-off score of 48 on global functioning was associated with a higher risk of engaging in disruptive behavior. Therefore, disruptive behavior could be predicted from patients’ global functioning from the very first day of hospitalization. If functioning remains poor or worsens, this is an additional signal for an increased risk of disruptive behavior. Consequently, there appears to be a window of opportunity for controlling disruptive behavior by improving patients’ global functioning. Improving patients’ global functioning would potentially lower their risk of engaging in disruptive behavior. Interventions for patients’ psychological and social functioning, violence risk, and ADL would appear to reduce the risk of the disruptive behavior from occurring. This proposal is consistent with the guidelines of HIC, which focus on recovery of patients’ health, daily functioning, societal roles, and identity. This is also consistent with the guidelines for patients with a psychotic disorder, who are the patients with a higher risk of displaying disruptive behavior. Additionally, greater attention should be given to early interventions that focus on the therapeutic relationship, patients’ ability to mentalize, their coping skills, group engagement, involvement in their treatment, and a daily program with sports and other daily living activities for improving patients’ global functioning and preventing potential disruptive behavior from occurring. Further research is needed to investigate the effects of these interventions on patients’ behavior.
